# The Impact of Probiotics and Egg Yolk IgY on Behavior and Blood Parameters in a Broiler Immune Stress Model

**DOI:** 10.3389/fvets.2020.00145

**Published:** 2020-04-09

**Authors:** Ibrahim F. Rehan, Mohammed Youssef, Mootaz A. M. Abdel-Rahman, Sohaila G. Fahmy, Eslam Ahmed, Ahmed S. Ahmed, Mohamed A. Maky, Hassan M. Diab, Obeid Shanab, Saad Alkahtani, Mohamed M. Abdel-Daim, Hamdy Hassan, Ahmed F. Rehan, Mohamed A. Hussien, Nesreen Z. Eleiwa, Asmaa Elnagar, Ahmed Abdeen, Abd El-Latif Hesham

**Affiliations:** ^1^Department of Husbandry and Development of Animal Wealth, Faculty of Veterinary Medicine, Menofia University, Shibin Alkom, Egypt; ^2^Department of Animal Physiology, Faculty of Veterinary Medicine, South Valley University, Qena, Egypt; ^3^Department of Behavior, Management, and Development of Animal Wealth, Faculty of Veterinary Medicine, Minia University, El-Minia, Egypt; ^4^Department of Animal Behavior and Management, Faculty of Veterinary Medicine, South Valley University, Qena, Egypt; ^5^Department of Food Hygiene and Control (Milk Hygiene), Faculty of Veterinary Medicine, South Valley University, Qena, Egypt; ^6^Department of Food Hygiene and Control (Meat Hygiene), Faculty of Veterinary Medicine, South Valley University, Qena, Egypt; ^7^Department of Animal and Poultry Health and Environment, Faculty of Veterinary Medicine, South Valley University, Qena, Egypt; ^8^Department of Biochemistry, Faculty of Veterinary Medicine, South Valley University, Qena, Egypt; ^9^Department of Zoology, College of Science, King Saud University, Riyadh, Saudi Arabia; ^10^Pharmacology Department, Faculty of Veterinary Medicine, Suez Canal University, Ismailia, Egypt; ^11^Department of Animal Production, Faculty of Agriculture, South Valley University, Qena, Egypt; ^12^Department of Food Control, Faculty of Veterinary Medicine, Zagazig University, Zagazig, Egypt; ^13^Department of Food Hygiene, Animal Health Research Institute, Agricultural Research Center, Giza, Egypt; ^14^Department of Biochemistry, Faculty of Veterinary Medicine, Zagazig University, Zagazig, Egypt; ^15^Department of Forensic Medicine and Toxicology, Faculty of Veterinary Medicine, Benha University, Toukh, Egypt; ^16^Department of Genetics, Faculty of Agriculture, Beni-Suef University, Beni-Suef, Egypt

**Keywords:** immunoglobulin Y, behavior, physiology, lipopolysaccharides, broilers

## Abstract

Feed additives are used frequently in variable combinations to maximize broiler productivity and consumer safety. Therefore, we evaluated the efficiency of feed additives used in four different diets: a basal diet, a probiotic (PRO-PAC^®^) supplement diet, an egg yolk purified immunoglobulin Y (IgY) supplemented diet, and a combination of IgY and PRO-PAC^®^ supplement (*n* = 15 for each group). We assessed the improvement of behavioral and hematological parameters of Ross broilers before and after an immune stress challenge using lipopolysaccharide (LPS). Behavioral as well as physiological parameters were analyzed. The standing frequency was the highest (*P* < 0.05) in broilers supplemented with a combination of probiotics and IgY. Likewise, latency approach score to a novel object improved (*P* < 0.01) in the combination group at week-3. After intraperitoneal injection of LPS, this combination group achieved the best gait score at week-3, followed by week-5, compared to birds fed the basal diet. The heterophil/lymphocyte (H/L) ratio, heterophil differential count, and eosinophil differential count in the basal diet group that was challenged with LPS were significantly increased (*P* < 0.01, *P* < 0.001, *P* < 0.05, respectively) compared to the combination groups. Therefore, we concluded that the combination of IgY and probiotics can significantly improve the behavior and the underlying physiological parameters of Ross broilers. Consequently, this combination can improve the broilers′ health, welfare and produce a safe meat free from harmful chemical residues.

## Introduction

The high demand for poultry meat promotes intensive growth of poultry production. However, this intensive production has always been challenged by numerous obstacles such as physical, environmental, and medical stressors (1). The inflammatory symptoms accompanied by the innate immune response are common stressors in poultry farms that can result in major economic losses (2). Antibiotics are used in poultry farms to enhance the growth and feeding efficiency, in addition to limiting infections (3). The disastrous complications of widespread use of antibiotics, including microbial resistance, and harmful residues in broiler meat have urged additional efforts to find out new alternatives to antibiotic use. Feed additives were able to fulfill this gap through interactions with gut microbiota and contributions to the health of the host (4). Using probiotics as feed additives can initiate resistance to bacterial colonization, and promote the host mucosal immunity for minimizing pathogen load (5).

Egg yolk plasma contains immunoglobulins that have been widely used as biomedical and therapeutic products for controlling alimentary tract diseases (6). Hen immunoglobulins are naturally transferred to offspring through the egg yolk, which contains 8–20 mg of immunoglobulin Y (IgY)/ml (7, 8). The main function of IgY is to enhance the resistance of the developing embryo until the individual is fully capable of producing antibodies. IgY technology, in other words the production and use of polyclonal IgY (9), is considered to be a potential substitution for conventional polyclonal antibody production in mammals. The use of hen's egg yolk IgY as a feed additive has numerous advantages that involve (i) making of preferential use of chicken products, (ii) addressing the global challenge of microbial drug-resistant through reduction of antibiotic usage in the livestock industry, (iii) stimulation of immunogenicity against conserved mammalian proteins, which exists in birds due to their phylogenetic distance (10), which allows laying hens to be more efficient generators of antibodies against conserved epitopes compared to other animals, and (iv) providing passive vaccination against some bacterial infections, including *Salmonellosis, Campylobacteriosis, Staphylococcus aureus, Streptococcus suis*, and *Brucella* spp. (11). Moreover, IgY is a y-shaped antibody with the typical light and heavy chains structure that is similar to mammalian IgG. Also, the structure of the IgY fragment crystallization (FC) region does not allow strong binding to FC receptors on immune cells of non-avian species such as mice, which reduces associated inflammatory signs and allergic reactions when IgY is used for passive immunization in mammals (12).

Consequently, scientists have paid more attention to efficient purification of avian antibodies (13). The water dilution (WD) method that was described previously (14) is a fast and simple method to purify IgY from whole egg yolk. WD can provide the highest yield of antibodies (96%) while remaining cost-effective (15). During the first 2 weeks posthatching, the chick's adaptive immune system begins to develop. Meanwhile, the early humoral protection in the chick relies heavily on the maternal transfer of antibodies (16). Feeding the specific IgY purified from egg yolk to offspring is considered to be a continuation of passive maternal protection. Moreover, the production of large quantities of IgY in a cost-effective manner is essential for producing passive immunization in the broiler industry. Most research articles discussing IgY stability were done *in vitro*, and therefore, numerous studies are needed to assess IgY stability in the diet (17) for future efficient commercial applications.

The novel object test was performed at week-3 and week-5. This test was used to evaluate birds' reactions to a novel stimulus to detect whether the diet can affect the responsiveness of the broilers. In the current study, broiler stress model was established via lipopolysaccharide (LPS) challenge that is used frequently to induce immune stress and for simulation of animal response to infection. LPS exaggerates plasma corticosterone levels as an immediate stress response (18). LPS is a structural content of the outer wall of gram-negative bacteria (19), which acts as a toll-like receptor agonist and triggers the inflammatory signaling in immune cells, leading to oxidants and proinflammatory cytokines production that mediates the innate immune response (20, 21). The symptoms manifested by challenged birds include appetite loss, lack of activity, and high temperature at least within the first 4 h after LPS injection. LPS induces leukocytosis in challenged birds because of the initial elevation of the heterophil count and the heterophil/lymphocyte ratio within the first 24 h (22). The symptoms manifested by challenged birds include appetite loss, lack of activity, and high temperature at least within the first 4 h after LPS injection. LPS induces leukocytosis in challenged birds because of the initial elevation of the heterophil count and the heterophil/lymphocyte ratio within the first 24 h (22).

LPS also has a drastic impact on intestinal epithelium represented by retardation of epithelial cell growth and proliferation, which in turn hinders the absorption process (23). Using direct-fed microbial products such as *B. subtilis*-based probiotics in LPS-challenged chickens can significantly reduce systemic inflammation and decrease serum levels of acute-phase glycoproteins such as α1-acid glycoprotein (24). The same author also disclosed the ameliorative impact of probiotics against LPS-induced inflammation that is emphasized by reduction in LPS-induced proinflammatory cytokines, and revealed that probiotics can also modulate intestinal immune activities, and potentially stabilize gut integrity during inflammation. However, as far as we know, the available data evaluating the effect of IgY combined with probiotics on the health and welfare of broilers in normal and immune stress conditions are not clear and scarce. Therefore, we hypothesized that using probiotics along with IgY can potentially enhance broilers' immune system and contribute greatly to the improvement of poultry management as well as safe meat production. Therefore, this study was performed to investigate the effect of natural feed additives (IgY, probiotics, and a mixture of both), on promoting behavioral activities, health status, and immunity in broilers exposed to LPS.

## Materials and Methods

### Ethical Approval

We tried to minimize the discomfort of the chicks as much as possible during the study period. We did not rear the birds during rain, hot, or cold weather. Only workers and relevant researchers could enter the birds' room. The experiment was approved, and the birds were identified and managed under license number (161411-04-2018) from the Animal Ethics Committee of the Faculty of Vet-Med., South Valley University, Qena, Egypt.

### Accommodation (Birds, Housing, and Management)

All manure was scrubbed from the poultry house. In addition, dust and dirt were removed from windows, floors, ceiling, walls, and ventilation shafts using water and high-pressure sprayers, and then the surfaces of the housing units were disinfected using Formalin^®^ 35–38% (5/100). Utensils and removable equipment were soaked in the same disinfectant solution. Moreover, fumigation disinfection was also applied. Finally, quicklime was sprayed on the ground under the litter. The four pens were 3.9 m^2^ (1.8m L × 2.2m W × 3m H). The daily management protocol for the broilers (lighting, temperature, relative humidity, and vaccination program) was carried out as previously reported (25). Birds had access to feed and water *ad-libitum*. The commercial feed used during this experiment was purchased from Alaaf ALmagd, Alarabia Lell-alaaf, Quesna, Menuofia, Egypt. The chemical composition of the diet was prepared according to the previous report (26). A commercial probiotic mixture, PRO-PAC^®^ (Nutrivet Animal Health. Co. LTD, Egypt), was used. Each kg of PRO-PAC^®^ was composed of 100 g of Betaine HCl 97%, 100 g of *Lactobacillus acidophilus*, 50 g of *Enterococcus faecium*, 4.8 g of *Lactobacillus Plantarum*, 2 g of *Bifidobacterium bifidum*, 50 g of *Aspergillus oryzae* fermentation extracts (xylanase 12,500 units/kg, hemicellulase 2,750 units/kg, and ß-glucanase 2,250 units/kg), and 50 g/kg *Bacillus subtilus* fermentation extracts (alpha aylase 25,000 units/kg, cellulose 4,500 units/kg, and protease 12,500 units/kg). Preparation of IgY from egg yolk was performed using the “water dilution” method (14). After the preparation of egg yolk, the mixture was precipitated using ammonium sulfate as previously reported (27). Finally, we obtained IgY in a powder form, as mentioned previously (28).

### Experimental Design

Sixty-one-day-old non-sexed broiler chicks (Ross) were randomly divided into four groups (15/group). In each group, five chicks were marked with different colors on their head and back for behavioral observation. Another five birds were used for the blood sampling, and the remaining birds in the same group were kept as spares. The first group (control group) of broilers was fed the basal diet. The second group (probiotic group) of broilers was fed the basal diet supplemented with PROPAC^®^ (0.5 g/kg diet). The third group (IgY group) was broilers fed the basal diet supplemented with IgY powder (0.5 g/ kg diet). The fourth group (the combination group) was broilers supplemented with a mixture of IgY and probiotics (0.25 g/kg diet each). Meanwhile, probiotic was added from day-1 to day-42 of age, while IgY was added from day-8 to day-42 of age. The stress model was performed at day-28 of age, and each treatment group of broilers was further subdivided into two subgroups with four birds each randomly chosen. LPS was diluted in physiological saline and injected intraperitoneally at a dose of 0.1 mg/kg of body weight. Blood samples were collected 3 h later, from the wing vein of all 32 experimental birds using EDTA (1 mg ml^−1^) as an anticoagulant (29). Fresh samples were used for assessment of hemoglobin (Hb) concentration, the ratio of packed cell volume (PCV), and differential leukocyte count (DLC), as described previously (30).

### Behavior and Welfare Assessments

#### Behavioral Observation

Behavioral activities of each group were observed once weekly (three times/day) from week-3 to week-6. Behaviors were recorded for 30 min in three interval periods (9 AM, 1 PM, and 5 PM) (31), at days 21,28,35, and 42 of age using a computerized camera recording system. The cameras (Hikvision, Binjiang District, Hangzhou, China) were fixed, directly overhead, and recorded in real time. Data were stored on Hikvision-DVRs for the behavioral analysis.

#### Ethogram (Time-Budgets)

All birds' activities were obtained from the 30 min scans at 5 min intervals. The behavioral patterns such as standing activity and lying were recorded. The standing activities as stand, preen stand, stand to eat, peck body, peck head and/or litter, drink, walk, jump, and run were recorded. The behavioral patterns of lying position as preen lie, lie eat, lie sleep, leg stretch, wing stretch, leg and wing stretch, and wing flap were displayed, as previously reported.

#### Novel Object Test

The novel object was different in shape and color at week-3 compared to week-5 (32). The results of these tests were collected by calculating the latency approach (LA) score, and latency peck (LP) score, using the following formula:

LA score = [LA to the object (s)/total duration of test (s)] × 100. However, LP score = [LP to the object (s)/ total duration of test (s)] × 100.

#### Gait Score (GS)

The gait score was calculated at week-3 and week-6. The gait score is considered an indicative sign in broilers, as described previously (33). For instance (0) = its gait is smooth and the foot curls when lifted, and the bird appears well-balanced; (1) = its gait is uneven, the foot may or may not curl when lifted, and it is difficult to tell which side has the injured leg or foot; (2) = its gait is uneven, the foot remains flat when lifted, the bird's stride is shortened, and the bird may have poor balance and use its wings for support; (3) = similar to gait score of 2 but remains lying down unless gently nudged to move, more likely to use wings for balance and support; also, it cannot stand for more than 25 s and typically lies down after a series of steps; (4) = it is reluctant to move up to 5 s before the bird stands on both feet, and uses wings like crutches to walk, and the bird can only take a few steps before lying back down; (5) = it is not able to take one step, and will shuffle if nudged to move.

### Euthanasia and Blood Collection

The birds were euthanized on day-42 of age by decapitation technique. Blood (5 ml) was collected in tubes containing k3-EDTA as an anticoagulant. Another 5 ml was collected in vacuum tubes without EDTA to obtain sera. Feed was withdrawn 6–8 h before euthanasia (34). During euthanasia, caution was taken, as much as possible, to reduce animal stress.

Blood smears were prepared using fresh samples (two replicates from each sample). The collected blood samples were cooled to approximately 4°C using icepacks, and were transferred to the laboratory within 2 h after blood collection. Blood in tubes without EDTA was used to obtain sera by centrifugation at 1,500 rpm for 15 min (22). The collected sera were kept at −20°C until biochemical analysis.

### Blood Parameters

#### Packed Cell Volume (PCV)

PCV values were determined, as described before (35), with slight modifications.

#### Hemoglobin (Hb) Concentration

Quantitative Hb determination, purchased from Vitro scientific, Egypt, was carried out using a commercial colorimetric kit (36).

#### Differential Leukocytic Counts (DLC)

Cell counts were calculated and then categorized according to their morphology and the ratio of each cell type. The heterophil/lymphocytes ratio (H/L) was determined as an indicator of stress (37).

#### Estimation of Mortality Rate

The number of mortalities was recorded daily during the entire experiment in control as well as treatment groups.

### Statistical Analysis

All statistical steps were carried out using SPSS-software, version 16. Data were analyzed using one-way-ANOVA with the general linear models procedure. The comparison of means was done using Duncan's multiple range tests. Differences between means were due to the different groups. Data were written as mean ± SEM, and the differences were considered significant at *P* < 0.05.

## Results

No significant differences in behavioral activities were observed in week-3 (Tables S1, S2). We predicted that the activities and rests of the birds would be started abundantly from week-4. Therefore, the behavioral patterns of the broilers were more intensely observed with the advancement of age. Moreover, the duration of standing activities (s) was the highest in broiler group supplemented with probiotics mixed with IgY at week-4 [1343.2 ± 80.9, F_(3, 16)_ = 1.539, *P* = 0.032], week-5 [624.7 ± 22.3, F_(3, 16)_ = 1.721, *P* = 0.006], and week-6 [713.8 ± 44.4, F_(3, 16)_ = 1.743, *P* = 0.002], respectively (see Tables S5, S9, S13). The standing frequency (bout/30 min) was the highest in the group supplemented with probiotics plus IgY in week-3 (F_3,16_ = 1.643, *P* = 0.041) and week-5 (F_3,16_ = 1.823, *P* = 0.004). However, the group supplemented with IgY in week-4 and week-6 had no significant difference (see Tables S3, S7, S11, S15).

The duration of lying condition/rest (s) was the lowest in the group supplemented with probiotics plus IgY at week-4 [456.7 ± 67.9, F_(3, 16)_ = 1.723, *P* = 0.024] and week-6 [921.7 ± 48.3, F_(3, 16)_ = 1.628, *P* = 0.01] (see Tables S6, S14). Although the duration of the lying condition tended to be the lowest in the same group at week-5 (740.5 ± 81.1 s), there is no significant difference (Table S10). The lying frequency presented no significant differences from the 3^rd^ ~ 6^th^ week of age in all groups (see Tables S4, S8, S12, S16). The average standing frequency over the entire production period was the highest [46.8 ± 2.17 bouts, F_(3, 16)_ = 1.617, *P* < 0.05] in broilers supplemented with a combination of probiotics and IgY (Table 1). The mortalities recorded 13% in a control group but no records found in other treatment groups (see Table S17).

**Table 1:** The total standing and lying (time “s,” and frequency “bout,” mean ± SEM) of broilers supplemented with different feed additives during the production period (week-3 ~ week-6).

**No**.	**Group**	**Standing activity time**	**Lying time**	**Frequency of activity**	**Frequency of lying**
1	Control	823.1 ± 48.08	898.2 ± 2.02	34.1 ± 2.02	20.47 ± 0.82
2	Probiotic	805.8 ± 29.03	1041.3 ± 44.36	41.6 ± 1.45	19.85 ± 0.49
3	IgY	640.2 ± 33.3	1197.8 ± 38.16	33.5 ± 1.14	21.67 ± 0.76
4	Probiotic + IgY	792.2 ± 38.8	1147.8 ± 43.13	46.8 ± 2.17^*^	22.20 ± 0.7

*Data were presented as mean ± SEM and analyzed using one-way-ANOVA. IgY, immunoglobulin Y*;

* *P < 0.05*.

As shown in Table 2, the LA scores to the novel object were significantly increased from week-3 [3.75 ± 2.7, F_(3, 16)_ = 1.723, *P* = 0.005] to week-5 [0.89 ± 0.32, F_(3, 16)_ = 1.842, *P* = 0.004] in broiler groups supplemented with a mixture of probiotic and IgY. The LP score to the object was recorded only in the broilers supplemented with probiotics at week-5 (6.04 ± 0.5).

**Table 2:** The latency of approach and peck at the 3rd week and 5th week of the broiler groups supplemented with different feed additives.

	**Group**	**LA (week-3)**	**LA (week-5)**	**LP (week-3)**	**LP (week-5)**
1	Control	1.35 + 0.33	0.48 + 0.14	—	—
2	Probiotic	2.55 + 0.90	0.22 + 0.07	—	6.04 ± 0.5
3	IgY	0.82 + 0.17	0.30 + 0.11	—	—
4	Probiotic + IgY	3.75 + 2.7^**^	0.89 + 0.32^**^	—	—

*Data were presented as mean ± SEM and analyzed using one-way-ANOVA. IgY, immunoglobulin Y; LA, latency of approach; LP, latency of pecking; —, not detected*;

** *P < 0.01*.

The results shown in Table 3 indicated that the control group was the only group in a poor GS (0.2 ± 0.20) at week-3. The score for walking disabilities (1.2 ± 0.24) increased in the same group at week-5. However, the broilers group supplemented with IgY and probiotics was the best to show the ideal GS (zero) at week-3, and (0.2 ± 0.20) at week-5.

**Table 3:** The gait score at the 3rd and 5th week of broiler supplemented with different feed additives and after challenge with LPS.

**No**.	**Group**	**GS (week-3)**	**GS (week-5)**	**GS (week-5) after LPS-challenge**
1	Control	0.2 ± 0.20	1.2 ± 0.24	1.7 ± 0.63
2	Probiotic	0	1.0 ± 0.31	1.2 ± 0.37
3	IgY	0	0.4 ± 0.49	0.6 ± 0.24
4	Probiotic + IgY	0	0.2 ± 0.20	0.4 ± 0.24

*Data were presented as mean ± SEM and analyzed using one-way-ANOVA. GS: gait score; IgY: immunoglobulin Y; LPS: lipopolysaccharide. The gait score (0~5) is considered an indicative signs in broilers, as described previously (33)*.

The RBC counts from the control group that was challenged with LPS were the highest (212 ± 10.58), and the counts from the IgY-supplemented group that was challenged with LPS were the lowest (180 ± 6.92). However, the Hb concentration in all groups showed no significant differences. In addition, the PCV ratio in the IgY group, challenged with LPS, was lower [30 ± 1.15, F_(7, 24)_ = 1.959, *P* = 0.021] compared to the control group (35.33 ± 1.76, *P* = 0.006), and in the probiotic group (33.66 ± 1.76, *P* = 0.032), (see Table 4).

**Table 4:** Blood analysis of broilers supplemented with different feed additives.

**No**.	**Group**	**Blood analysis**		
		**RBCs**	**Hb concentration**	**PCV%**
1	CNT-Saline	190 ± 5.29	15.11 ± 1.36	31.66 ± 0.88
2	CNT-LPS	212 ± 10.58	17.78 ± 1.25	35.33 ± 1.76^**^
3	Probiotic-Saline	202 ± 10.58	15.54 ± 1.00	33.66 ± 1.76^*^
4	Probiotic-LPS	204 ± 3.46	16.42 ± 1.42	34 ± 0.57
5	IgY-Saline	190 ± 7.21	15.32 ± 1.29	31.66 ± 1.2
6	IgY-LPS	180 ± 6.92	14.5 ± 1.73	30 ± 1.15^*^
7	IgY plus Probiotic-Saline	183 ± 1.73	16.19 ± 1.43	30.5 ± 0.28
8	IgY plus Probiotic-LPS	182 ± 14.0	14.45 ± 2.65	30.33 ± 2.33

*Data were presented as mean ± SEM and analyzed using one-way-ANOVA. CNT, control group; RBCs, red blood cell counts; Hb, hemoglobin; PCV, packed cell volume; LPS, control group challenged with LPS; LPS, lipopolysaccharides; IgY, immunoglobulin Y; —, not detected*;

* *P < 0.05*;

** *P < 0.01*.

As shown in Table 5, the H/L ratio [1.5 ± 0.31, F_(4, 18)_ = 1.324*, P* = 0.006], heterophils % [51.57 ± 4.84, F_(4, 18)_ = 1.537, *P* = 0.001], and eosinophils % [1.14 ± 0.40, F_(4, 18)_ = 1.433, *P* = 0.03] were increased in the broiler control group challenged with LPS, in comparison to the IgY-supplemented group. Lymphocyte % was significantly decreased [39.14 ± 3.99, F_(4, 18)_ = 1.440, *P* = 0.001] in the control group challenged with LPS, compared to the other groups. We noticed that basophils % and monocytes % presented no significant differences among all the groups.

**Table 5:** Differential leukocytic counts of broilers supplemented with different feed additives.

**No**.	**Group**	**(H/L) ratio**	**Heterophils**	**Lymphocytes**	**Basophils**	**Monocytes**	**Eosinophils**
1	CNT-Saline	0.51 ± 0.11	27.5 ± 5.01	64.75 ± 4.49	0.25 ± 0.16	6.5 ± 1.22	1.0 ± 0.59
2	CNT-LPS	1.5 ± 0.31^**^	51.57 ± 4.84^***^	39.14 ± 3.99	0.86 ± 0.40	7.28 ± 1.58	1.14 ± 0.40^*^
3	Probiotics	0.94 ± 0.16	43.42 ± 4.72	50.42 ± 4.13	0.14 ± 0.14	5.85 ± 1.24	0.14 ± 0.14
4	IgY	1.04 ± 0.26	42.37 ± 4.84	47.75 ± 4.25	0.5 ± 0.26	8.375 ± 1.41	1.0 ± 0.46
5	Probiotics plus IgY	0.69 ± 1.35	39 ± 8.62	57.66 ± 8.64^***^	0.0 ± 0.34	3.33 ± 0.94	0.0 ± 0.16

*Data were presented as mean ± SEM and analyzed using one-way-ANOVA. CNT, control group; (H/L), heterophils/lymphocytes; LPS, control group challenged with LPS; LPS, lipopolysaccharides; IgY, immunoglobulin Y; —, not detected*;

* *P < 0.05*;

** *P < 0.01*;

*** *P < 0.001*.

## Discussion

Although the beneficial effect of IgY as a supplement was reviewed before (17), the literature on the potential augmentation or depletion of IgY effect when combined with probiotics in stressed birds is scarce. Herein, we determined that addressing this aspect will clarify the impact of this combination on broilers' health and meat safety as reflected by behavioral and hematological parameters. Probiotic diet supplementation is an efficient and safe approach for prophylaxis against bacterial infection (38). However, a more effective and low-cost alternative is achieved through supplementation of polyclonal antibodies in the broiler's diet. IgY is the most prevalent immunoglobulin in avian serum with a more intense pathogen-specific response compared to mammalian IgG (39). Purification of IgY from laying hens' egg yolk is cost-efficient in large-scale production (17). Although IgY is provided naturally to offspring through egg yolk, it remains active only for about 2 weeks after egg hatching. Subsequently, the level of IgY in the chick's plasma decreases considerably (40). Therefore, non-invasive oral administration of IgY is necessary to maintain a high antibody titer. The favorable effect of IgY on broilers' health shown in the current study can be referred to the intact chemical structure of serum IgY, since ingested IgY is bioavailable in serum for up to 24 h, highly stable at 60 to 65 °C, and resistant to proteolytic activity of trypsin and chymotrypsin (41). Moreover, the addition of certain carbohydrates to IgY preparations increases the antibody stability up to 95°C and during the use of steam in the pelleting processing (42).

There were no differences in the behavioral activities at week-3. We predicted that the activities and rests of birds would be increased starting at week-4. The time standing activities like preen stand, stand, stand to eat, peck body, peck head, peck litter, drink, walk, jump, and run were highest in the broiler group supplemented with probiotics mixed with IgY at week-4, week-5, and week-6. Meanwhile, the standing frequency was the highest in broiler supplemented with a combination of probiotics and IgY. In addition, the standing frequency was the highest in the group supplemented with probiotics mixed with IgY in week-3 and in the group supplemented with IgY. Additionally, it was increased in the group supplemented with IgY in week-4, and in week-6. However, the duration of lying resting like preen lie, lie eat, lie sleep, leg stretch, wing stretch, leg and wing stretch, and wing flap was the lowest in the group supplemented with probiotics and IgY at week-4, and week-6.

Our results were in line with Iraqi (43) who found that feeding behavior, both its frequency and duration, was significantly increased in birds supplemented with probiotics, compared to the controls. Moreover, within the same study, with respect to preening behavior, the control group recorded the highest frequency and duration, followed by the live yeast group, which in turn was followed by the Thepax^®^ group. Furthermore, they also reported that resting behavior in the control group was significantly higher than in Thepax^®^ group. Likewise, the improvement of feed intake by IgY supplementation was emphasized recently in birds that received an aflatoxin-contaminated diet (44).

On the other hand, it was reported that probiotics had no significant impact on feeding frequency, feeding duration, preening, resting, fighting, and feather pecking behaviors, compared to the control group (45). Moreover, some previous results indicated no improvement in feed intake in probiotic-supplemented chicks (46). These negative results might be partially due to different probiotic products or the doses used in the diet formulation. Our results suggested that the passive immunization was critical to display the bird's fundamental needs in performing the essential behavioral patterns and to ensure the welfare.

The highest LA score to the novel object was increased in week-3, and week-5 for the broiler group supplemented with a mixture of probiotics and IgY. This result was in line with a previous investigation (43). However, lying was increased in broilers, associated with a fearful condition, and with suppression of the physiological responses (47). In our result, the behaviors of birds were essential for maintenance and survival in both starter and grower phases. However, the lying conditions for the two consequent weeks during the finishing phase might indicate fear or stress condition in birds, as these behaviors were reduced in the control group fed the basal diet only.

The GS of broilers was recorded at week-3 and week-5 to evaluate the walking ability. It is well-known that a lower GS is associated with increased walking abilities recorded in chickens. The result observed in this study indicated that the control group was the only group with a poor condition score at week-3. Meanwhile, the score of walking disabilities increased in the same group at week-5. However, the chickens supplemented with IgY mixed with probiotics were the best to show the ideal GS (zero) at week-3, and a good score at week-5. To confirm the walking abilities and the fitness of the locomotor system as the birds aged, we tested the GS of broilers after intraperitoneal injection of LPS. Challenging with LPS is used as a stress model due to the ability of LPS to elevate the serum stress hormone, corticosteroid, for several days after injection, in addition to the decline of feed ingestion (48, 49). Here, we showed that birds supplemented with IgY and probiotics were able to walk well at week-5 after the LPS challenge. However, the ability to walk was attenuated as the birds aged. Moreover, the broilers supplemented with IgY and probiotics achieved the best GS scores. Therefore, the combination of feed additives (IgY and probiotics) can clearly enhance the locomotor system of birds, which is reported to have a positive impact on their immunity (50).

Investigating the hematological parameters revealed a significant increase in the PCV value due to LPS-induced immune stress. The elevated PCV value was also associated with a trend toward but non-significant elevation in the RBC count and hemoglobin concentration, which is in accord with other reports (22). LPS treatment showed no significant response in these parameters above in the group supplemented with a combination of probiotics and IgY. This confirmed that supplementation of IgY with probiotics can cancel the effect of the LPS challenge. Moreover, LPS increased the H/L ratio in birds receiving a standard diet. On the other hand, LPS failed to change the H/L ratio in groups supplemented with IgY, probiotics, or both, which is consistent with a previous report that showed a decline in LPS-induced avian H/L ratio with IgY or probiotic supplementation (45, 51–53). Elevation of the H/L ratio, heterophil differential counts, and eosinophil differential counts in the birds challenged with LPS is considered to be due to the binding of LPS to toll-like receptor 4 on immune cells. This binding promotes proinflammatory cytokines production, including TNF-α, TNF-γ, and IL-6, followed by induction of heterophil proliferation in the innate immune response (54). The ability of IgY to decrease the acute elevation of the immune cell count might help to avoid the undesirable effects of inflammation while maintaining a passive immunization. Suppression of immune cell proliferation by IgY is a short-term action, since passive immunization with multiple maternal antibodies, provided through non-specific purified IgY, was reported to temporarily suppress the natural inflammatory response associated with infection (55). However, this effect is not consistent with probiotic supplementation, since other reports showed significant variation among probiotics groups, and the untreated groups fed a basal diet regarding lymphocyte and heterophil differential counts as well as the H/L ratio (56–58).

Noticeably, the minimum mortalities and best health conditions were recorded in the broiler groups supplemented with IgY, compared to the other groups, which also agreed with previous literature (59).

## Conclusion

Although antibiotics are used in chicken to enhance their growth and feed efficiency and limit disease occurrence, consumers are threatened with increased risk of harm from drug residues in broiler meat. Seeking a new alternative to antibiotic use is needed to produce safe poultry meat for human consumption. Therefore, we strongly recommend using IgY-powder in combination with probiotics as an immune-effective feed additive in poultry farms instead of harmful antibiotics.

## Data Availability Statement

The corresponding author(s) have access to all the data represented in this article, which is available on reasonable request.

## Ethics Statement

The animal study was reviewed and approved by The Animal Ethics Committee of the Faculty of Vet-Med., South Valley University, Qena, Egypt.

## Author Contributions

IR, MY, and MA-R contributed equally to the design and concept for this research and scientific paper. EA and SF provided the chemical materials used in the study. ASA, MM, HD, OS, SA, MA-D, HH, AR, MH, NE, AE, AA, and AH carried out the experimental procedures and analysis. All authors participated in all examinations of control and experimental groups. They also participated in the manuscript's writing and revisions. All authors have read and approved the final manuscript.

### Conflict of Interest

The authors declare that the research was conducted in the absence of any commercial or financial relationships that could be construed as a potential conflict of interest.

## References

[1] Farghly MFA Mahrose KM Galal AE Ali RM Ahmad EAM Rehman ZU. Implementation of different feed withdrawal times and water temperatures in managing turkeys during heat stress. Poult Sci. (2018) 97:3076–84. 10.3382/ps/pey173 29788365

[2] Shah M Zaneb H Masood S Khan RU Mobashar M Khan I Single or combined applications of zinc and multi-strain probiotic on intestinal histomorphology of broilers under cyclic heat stress. Probiotics Antimicrob Proteins. (2019) 1:1–8. 10.1007/s12602-019-09561-6 31154611

[3] Ohimain EI Ofongo RTS The effect of probiotic and prebiotic feed supplementation on chicken health and gut microflora: a review. Int J Anim Vet Adv. (2012) 4:135–43. Available online at: https://maxwellsci.com/jp/abstract.php?jid=IJAVA&no=195&abs=11

[4] Park JH Kim IH. Supplemental effect of probiotic *Bacillus subtilis* B2A on productivity, organ weight, intestinal *Salmonella* microflora, and breast meat quality of growing broiler chicks. Poult Sci. (2014) 93:2054–9. 10.3382/ps.2013-03818 24902699

[5] Sugiharto S Yudiarti T Isroli I Widiastuti E Kusumanti E Dietary supplementation of probiotics in poultry exposed to heat stress–a review. Ann Anim Sci. (2017) 17:591–604. 10.1515/aoas-2016-0062

[6] Trziszka T Saleh Y Kopeć W Siewinski M Wesierska E Effect of hen's age on the level of cystatin in the chicken egg white. Int J Poult Sci. (2004) 3:471–7. 10.3923/ijps.2004.471.477

[7] Batty I The transmission of passive immunity from mother to young. Immunology. (1971) 20:119.

[8] Yegani M Korver DR Application of egg yolk antibodies as replacement for antibiotics in poultry. Worlds Poult Sci J. (2010) 66:27–38. 10.1017/S0043933910000048

[9] Schade R Zhang XY Terzolo HR. Use of IgY antibodies in human and veterinary medicine. In: Bioactive Egg Compounds. Berlin; Heidelberg: Springer (2007) p. 213–22. 10.1007/978-3-540-37885-3_25

[10] Gassmann M Thömmes P Weiser T Hübscher U. Efficient production of chicken egg yolk antibodies against a conserved mammalian protein. FASEB J. (1990) 4:2528–32. 10.1096/fasebj.4.8.1970792 1970792

[11] Namata H Welby S Aerts M Faes C Abrahantes JC Imberechts H. Identification of risk factors for the prevalence and persistence of *Salmonella* in Belgian broiler chicken flocks. Prev Vet Med. (2009) 90:211–22. 10.1016/j.prevetmed.2009.03.006 19467722

[12] Abbas AT El-Kafrawy SA Sohrab SS Azhar EIA. IgY antibodies for the immunoprophylaxis and therapy of respiratory infections. Hum Vaccines Immunother. (2019) 15:264–75. 10.1080/21645515.2018.1514224 30230944 PMC6363154

[13] Schade R Chacana PA Livetin fractions (IgY). In: Bioactive Egg Compounds. Berlin; Heidelberg: Springer (2007) p. 25–32. 10.1007/978-3-540-37885-3_5

[14] Akita EM Nakai S. Production and purification of Fab′ fragments from chicken egg yolk immunoglobulin Y (IgY). J Immunol Methods. (1993) 162:155–64. 10.1016/0022-1759(93)90380-P 8315286

[15] De Meulenaer B Huyghebaert A Isolation and purification of chicken egg yolk immunoglobulins: a review. Food Agric Immunol. (2001) 13:275–88. 10.1080/09540100120094537

[16] Smith DP Lawrence KC Heitschmidt GW Fertility and embryo development of broiler hatching eggs evaluated with a hyperspectral imaging and predictive modeling system. Int J Poult Sci. (2008) 7:1001–4. Available online at: https://pubag.nal.usda.gov/catalog/40959

[17] Gadde U Rathinam T Lillehoj HS. Passive immunization with hyperimmune egg-yolk IgY as prophylaxis and therapy for poultry diseases – a review. Anim Heal Res Rev. (2015) 16:163–76. 10.1017/S1466252315000195 26568433

[18] Sternberg EM. Neural regulation of innate immunity: a coordinated nonspecific host response to pathogens. Nat Rev Immunol. (2006) 6:318–28. 10.1038/nri1810 16557263 PMC1783839

[19] Ulevitch RJ Tobias PS. Receptor-dependent mechanisms of cell stimulation by bacterial endotoxin. Annu Rev Immunol. (1995) 13:437–57. 10.1146/annurev.iy.13.040195.002253 7542010

[20] Tan J Liu S Guo Y Applegate TJ Eicher SD. Dietary L-arginine supplementation attenuates lipopolysaccharide-induced inflammatory response in broiler chickens. Br J Nutr. (2014) 111:1394–404. 10.1017/S0007114513003863 24330949

[21] Youssef M Ibrahim A Akashi K Hossain MS. PUFA-plasmalogens attenuate the LPS-induced nitric oxide production by inhibiting the NF-kB, p38 MAPK and JNK pathways in microglial cells. Neuroscience. (2019) 397:18–30. 10.1016/j.neuroscience.2018.11.030 30496826

[22] Xie H Rath NC Huff GR Huff WE Balog JM. Effects of *Salmonella typhimurium* lipopolysaccharide on broiler chickens. Poult Sci. (2000) 79:33–40. 10.1093/ps/79.1.33 10685886

[23] Hu X Guo Y Li J Yan G Bun S Huang B Effects of an early lipopolysaccharide challenge on growth and small intestinal structure and function of broiler chickens. Can J Anim Sci. (2011) 91:379–84. 10.4141/cjas2011-008

[24] Gadde UD Oh S Lee Y Davis E Zimmerman N Rehberger T. Dietary Bacillus subtilis- based direct-fed microbials alleviate LPS-induced intestinal immunological stress and improve intestinal barrier gene expression in commercial broiler chickens. Res Vet Sci. (2017) 114:236–43. 10.1016/j.rvsc.2017.05.004 28505587

[25] Ahmed E Abdelrahman M Gahreeb K Effect of probiotic on growth performance, carcass traits, and clinical health parameters of broilers reared under heat stress in upper Egypt. SVU-Int J Vet Sci. (2019) 2:27–44. 10.21608/svu.2019.11221.1012

[26] National Research Council Science and Judgment in Risk Assessment. Washington, DC: The National Academies Press (1994). 10.17226/2125 25032408

[27] Schwarzkopf C Thiele B. Effectivity of different methods for the extraction and purification of IgY. ALTEX. (1996) 13:35–39. 11178470

[28] Jaradat ZW Marquardt RR Studies on the stability of chicken IgY in different sugars, complex carbohydrates and food materials. Food Agric Immunol. (2000) 12:263–72. 10.1080/09540100020008137

[29] Tayeb I Qader G Effect of feed supplementation of selenium and vitamin E on production performance and some hematological parameters of broiler. KSÜ Doga Bil Derg. (2012) 15:46–56. Available online at: https://dergipark.org.tr/tr/pub/ksudobil/issue/22834/243778

[30] Hoffmann PR. Mechanisms by which selenium influences immune responses. Arch Immunol Ther Exp (Warsz). (2007) 55:289–97. 10.1007/s00005-007-0036-4 18219759

[31] Arnould C Faure JM Use of pen space and activity of broiler chickens reared at two different densities. Appl Anim Behav Sci. (2004) 87:155–170. 10.1016/j.applanim.2004.01.001

[32] Keer-Keer S Hughes BO Hocking PM Jones RB Behavioral comparison of layer and broiler fowl: measuring fear responses. Appl Anim Behav Sci. (1996) 49:321–33. 10.1016/0168-1591(96)01055-6

[33] de Jong IC Hindle VA Butterworth A Engel B Ferrari P Gunnink H. Simplifying the welfare quality ^®^ assessment protocol for broiler chicken welfare. Animal. (2016) 10:117–27. 10.1017/S1751731115001706 26306882

[34] Roepstorff K Rasmussen I Sawada M Cudre-Maroux C Salmon P Bokoch G. Stimulus-dependent regulation of the phagocyte NADPH oxidase by a VAV1, Rac1, and PAK1 signaling axis. J Biol Chem. (2008) 283:7983–93. 10.1074/jbc.M708281200 18160398

[35] Benjamin MM Outline of Veterinary Clinical Pathology, 3rd Edn. Ames: The Iowa State University Press (1979).

[36] Franco RS Hemoglobin. In: Kaplan LA Pesce AJ Kazmierczak SC, editors. Clinical Chemistry. St Louis, MO; Toronto, ON; Princeton, NJ: The CV Mosby Co (1984) p. 1294–6.

[37] He SP Arowolo MA Medrano RF Li S Yu QF Chen JY Impact of heat stress and nutritional interventions on poultry production. World Poultry Sci J. (2018) 74:647–64. 10.1017/S0043933918000727

[38] Carey CM Kostrzynska M. Lactic acid bacteria and bifidobacteria attenuate the proinflammatory response in intestinal epithelial cells induced by *Salmonella* enterica serovar Typhimurium. Can J Microbiol. (2013) 59:9–17. 10.1139/cjm-2012-0446 23391223

[39] Zhou X Ma S. Anti-lipopolysaccharide egg yolk antibodies enhance the phagocytosis of mammalian phagocytes. Biol Open. (2018) 7:bio.032821. 10.1242/bio.032821 29739752 PMC6031336

[40] Rehan IF Mohammed HH Fahmy SG Elnagar A Youssef M Shanab O Influence of photoperiod and circulating-IgY on some behavioral patterns of chicks during the first week of life. Int J Vet Sci AnimHusbandry. (2019) 4:18–25. Available online at: http://www.veterinarypaper.com/archives/2019/4/2/A/4-2-2

[41] Fink A Goose-Derived Igy: a potential therapeutic antibody for the treatment of infectious disease. Theses Diss. (2014) 1650 Available online at: https://commons.und.edu/theses/1650

[42] Bobeck EA Cook CL Gelbach BE Yang M Cook ME Thermoprotection of bioactive proteins added to animal feed. Worlds Poult Sci J. (2008) 64:199.

[43] Iraqi K Fayed R Effect of yeast as feed supplement on behavioral and productive performance of broiler chickens. Life Sci J. (2012) 9:4026–31. 10.7537/marslsj090412.600

[44] Nakhaei A Afzali N Hosseini Vashan SJM Karimi Torshizi MA To reduce the effects of experimental aflatoxicosis in broiler chicks using specific egg yolk immunoglobulin (IgY). Arch Med Lab Sci. (2019) 4:17–22. Available online at: http://journals.sbmu.ac.ir/archives/article/view/23058/18022

[45] Fayed RH Tony MA Effect of probiotic supplementation as anti-stress factor on growth performance, behavior and carcass traits of broiler chickens. Proc 1st Mediterr Summit WPSA, Porto Caras, Greece. (2008) 518–524.

[46] Javandel F Nosrati M van den Hoven R Seidavi A Laudadio V Tufarelli V. Effects of Hogweed (Heracleum persicum) powder, flavophospholipol, and probiotics as feed supplements on the performance, carcass and blood characteristics, intestinal microflora, and immune response in broilers. J Poult Sci. (2019) 56:262–9. 10.2141/jpsa.0180081 32055223 PMC7005398

[47] Mack LA Felver-Gant JN Dennis RL Cheng HW. Genetic variations alter production and behavioral responses following heat stress in 2 strains of laying hens. Poult Sci. (2013) 92:285–94. 10.3382/ps.2012-02589 23300291

[48] Shini S Kaiser P Shini A Bryden WL. Differential alterations in ultrastructural morphology of chicken heterophils and lymphocytes induced by corticosterone and lipopolysaccharide. Vet Immunol Immunopathol. (2008) 122:83–93. 10.1016/j.vetimm.2007.10.009 18045696

[49] O'Bryan MK Schlatt S Phillips DJ de Kretser DM Hedger MP. Bacterial lipopolysaccharide-induced inflammation compromises testicular function at multiple levels *in vivo*. Endocrinology. (2000) 141:238–46. 10.1210/endo.141.1.7240 10614644

[50] Chalghoumi R Beckers Y Portetelle D Thewis A Hen egg yolk antibodies (IgY), production and use for passive immunization against bacterial enteric infections in chicken: a review. Biotechnol Agron Société Environ. (2009) 13:295–308. Available online at: https://popups.uliege.be/1780-4507/index.php?id=4136

[51] Jeon IS Kang HK Kim CH Hwangbo J Park SB Effects of egg yolk antibody powder (IgY) supplementation on growth performance, blood component profile, intestinal microflora, and immunoglobulin G in meat ducks. Korean J Poult Sci. (2016) 43:143–8. 10.5536/KJPS.2016.43.3.143

[52] Mansoub NH Myandoab MP Comparative effect of using Zizaphora (Thymus valgaris), Garlic and probiotic on performance and serum composition of broiler chickens. Ann Biol Res. (2011) 2:373–8.

[53] Muhammad AA Effect of Drinking Water Supplementation With Probiotics and Prebiotics on Productive and Physiological Parameters of Broiler Chicks (Master thesis). Sulaimani University, Faculty of Agricultural Sciences, Sulaimaniyah, Iraq (2013).

[54] Rohde F Schusser B Hron T Farkašová H Plachý J Härtle S. Characterization of chicken tumor necrosis factor-α, a long missed cytokine in birds. Front Immunol. (2018) 9:605. 10.3389/fimmu.2018.00605 29719531 PMC5913325

[55] Abou Alazab MF Horiuchi H Furusawa S Induction of non-specific suppression in chicks by specific combination of maternal antibody and related antigen. J Vet Med Sci. (2015) 77:1363–9. 10.1292/jvms.14-0525 26050841 PMC4667651

[56] Mahmud M Shaba P James G Yisa HY Bello A Ndagimba R Comparative effects of supplementation of three probiotics on growth and haematological profiles of finishing broiler chickens. (2014).

[57] Owosibo AO Odetola OM Odunsi OO Adejinmi OO Lawrence-Azua OO Growth, haematology and serum biochemistry of broilers fed probiotics based diets. African J Agric. (2013) 8:5076–81. 10.5897/AJAR2013.7593

[58] Razek AHA Tony MA Effects of Dietary supplementation of a mixture of synbiotic and some digestive enzymes on performance, behavior and immune status of broiler chickens. Int J Anim Vet Adv. (2013) 5:75–81. 10.19026/ijava.5.5580

[59] Zuo Y Fan J Fan H Li T Zhang X. Prophylactic and therapeutic effects of egg yolk immunoglobulin against porcine transmissible gastroenteritis virus in piglets. Front Agric China. (2009) 3:104–8. 10.1007/s11703-008-0080-9 32214986 PMC7088736

