# Supplementary material for: The Impact of Probiotics and Egg Yolk IgY on Behavior and Blood Parameters in a Broiler Immune Stress Model
Source: Front Vet Sci. 2020 Apr 9;7:145. doi: 10.3389/fvets.2020.00145 (PMC7160245; doi:10.3389/fvets.2020.00145)
Supplement: Supplementary file 1 [file Data_Sheet_1.docx]

**SUPPORTING INFORMATION**

**The Impact of Probiotics and Egg Yolk IgY on Behavior and Blood Parameters in a Broiler Immune Stress Model**

***Ibrahim F. Rehan^1,*^, Mohammed Youssef^2,*^, Mootaz AM Abdel-Rahman^3^, Sohaila G. Fahmy^4^, Eslam Ahmed^4^, Ahmed S. Ahmed^5^, Mohamed A. Maky^6^, Hassan M. Diab^7^, Obeid Shanab^8^, Saad Alkahtani^9^, Mohamed M. Abdel-Daim^9,10^, Hamdy Hassan^11^, Ahmed F. Rehan^12^, Mohamed A. Hussien^12^, Nesreen Z. Eleiwa^13^, Asmaa Elnagar^14^, Ahmed Abdeen^15^, and Abd El-Latif Hesham^16^***

*^1^Department of Husbandry and Development of Animal Wealth, Faculty of Veterinary Medicine, Menofia University, Shebin Alkom, Menofia, 32511, Egypt.*

*^2^Department of Animal Physiology, Faculty of Veterinary Medicine, South Valley University, Qena, 83523, Egypt.*

*^3^Department of Behavior, Management, and Development of Animal Wealth, Faculty of Veterinary Medicine, Minia University, El-Minia, 61519, Egypt.*

*^4^Department of Animal Behavior and Management, Faculty of Veterinary Medicine, South Valley University, Qena, Egypt.*

*^5^Department of Food Hygiene and Control (Milk Hygiene), Faculty of Veterinary Medicine, South Valley University, Qena 83523, Egypt.*

*^6^Department of Food Hygiene and Control (Meat Hygiene), Faculty of Veterinary Medicine, South Valley University, Qena 83523, Egypt.*

*^7^Department of Animal and Poultry Health and Environment, Faculty of Veterinary Medicine, South Valley University, Qena 83523, Egypt.*

*^8^Department of Biochemistry, Faculty of Veterinary Medicine, South Valley University, Qena 83523, Egypt.*

*^9^Department of Zoology, College of Science, King Saud University, P.O. Box 2455, Riyadh 11451, Saudi Arabia.*

*^10^Pharmacology Department, Faculty of Veterinary Medicine, Suez Canal University, Ismailia 41522, Egypt.*

*^11^Department of Animal Production, Faculty of Agriculture, South Valley University, Qena 83523, Egypt.*

*^12^Department of Food Control, Faculty of Veterinary Medicine, Zagazig University, Zagazig, 44519, Egypt.*

*^13^Department of Food Hygiene, Animal Health Research Institute, Agricultural Research Center, Giza, Egypt.*

*^14^Department of Biochemistry, Faculty of Veterinary Medicine, Zagazig University, Zagazig 44519, Egypt.*

*^15^Department of Forensic Medicine and Toxicology, Faculty of Veterinary Medicine, Benha University, Toukh 13736, Egypt.*

*^16^Department of Genetics, Faculty of Agriculture, Beni-Suef University, Beni-Suef 62511, Egypt.*

***Correspondence****: Ibrahim F. Rehan, ibrahim.rehan@vet.menofia.edu.eg and Mohammed Youssef, mohamed.saleh@vet.svu.edu.eg.*

**TABLE S1** The statistics of standing activity time (sec/ 30 min) at 3^rd^ week of broilers supplemented with variant feed additives.

| **No.** | **Group** | **Preen stand** | **Stand** | **Stand eat** | **Peck body** | **Peck head** | **Peck litter** | **Drink** | **Walk** | **Jump** | **Run** | **Total** |
| --- | --- | --- | --- | --- | --- | --- | --- | --- | --- | --- | --- | --- |
| **1** | **Control** | 21.4 | 198.4 | 305.3 | --- | --- | 81.2 | 101.3 | 115.6 | --- | --- | 823.2±31.7 |
| **2** | **Probiotic** | 23.4 | 199.5 | 272.3 | 9.4 | --- | 70.3 | 84.5 | 139.3 | --- | 7.0 | 805.7±30.4 |
| **3** | **IgY** | 22.5 | 169.0 | 240.6 | 15.0 | --- | 21.6 | 109.9 | 61.7 | --- | --- | 640.3±27.4 |
| **4** | **Probiotic + IgY** | 12.0 | 177.0 | 340.3 | 3.8 | --- | 50.0 | 102.4 | 101.4 | --- | 5.3 | 792.2±36.2 |
| The mean time of behavioural patterns such as standing activity (e.g., preen stand, stand, stand eat, peck body, peck head, peck litter, drink, walk, jump and run) were recorded. Standing activity and lying time were presented as mean ± SEM. IgY: immunoglobulin Y; (---): not detected. | | | | | | | | | | | | |

**TABLE S2** The statistics of lying time (sec/ 30 min) at 3^rd^ week of broilers supplemented with variant feed additives.

| **No.** | **Group** | **Preen lie** | **Lie/Rest** | **Lie eat** | **Lie sleep** | **Leg stretch** | **Wing stretch** | **Leg/wing stretch** | **Wing flap** | **Total** |
| --- | --- | --- | --- | --- | --- | --- | --- | --- | --- | --- |
| **1** | **Control** | 32.2 | 721.1 | 121.0 | --- | 7.5 | 6.7 | 7.0 | 2.7 | 898.2±93.6 |
| **2** | **Probiotic** | 25.2 | 696.7 | --- | 299.9 | 2.8 | 5.8 | 5.5 | 5.5 | 1041.4±93.6 |
| **3** | **IgY** | 18.8 | 724.7 | 125.3 | 315.1 | --- | 5.5 | 4.2 | 4.3 | 1197.9±95.2 |
| **4** | **Probiotic + IgY** | 15.8 | 666.1 | 151.0 | 300.7 | --- | 4.2 | 5.9 | 4.1 | 1147.8±87.6 |
| The mean time of behavioural patterns such as rest/lying (e.g., preen lie, lie, lie eat, lie sleep, leg stretch, wing stretch, leg and wing stretch, wing flap) were recorded and analyzed using one-way-ANOVA. Standing activity and lying time were presented as mean ± SEM. IgY: immunoglobulin Y; (---): not detected. | | | | | | | | | | |

**TABLE S3** The statistics of frequency of activity (bout/ 30 min) at 3^rd^ week of broilers supplemented with variant feed additives.

| **No.** | **Group** | **Preen stand** | **Stand** | **Stand eat** | **Peck body** | **Peck head** | **Peck litter** | **Drink** | **Walk** | **Jump** | **Run** | **Total** |
| --- | --- | --- | --- | --- | --- | --- | --- | --- | --- | --- | --- | --- |
| **1** | **Control** | 3.2 | 5.0 | 5.6 | --- | --- | 5.0 | 3.7 | 11.6 | --- | --- | 34.1±0.9 |
| **2** | **Probiotic** | 2.6 | 7.4 | 4.0 | 3.1 | --- | 4.6 | 3.9 | 13.7 | --- | 2.5 | 41.8±1.2 |
| **3** | **IgY** | 3.3 | 7.4 | 3.9 | 1.8 | --- | 3.3 | 3.8 | 10.1 | --- | --- | 33.6±0.9 |
| **4** | **Probiotic + IgY** | 2.9 | 8.7 | 6.3 | 1.3 | --- | 4.8 | 5.0 | 16.1 | --- | 1.8 | 46.9±1.5* |
| The mean frequency of behavioural patterns such as standing activity (e.g., preen stand, stand, stand eat, peck body, peck head, peck litter, drink, walk, jump and run) were recorded and analyzed using one-way-ANOVA. Standing activity frequency was presented as mean ± SEM. IgY: immunoglobulin Y; (*), *P*< 0.05. (---): not detected. | | | | | | | | | | | | |

**TABLE S4** The statistics of frequency of lying (bout/ 30 min) at 3^rd^ week of broilers supplemented with variant feed additives.

| **No.** | **Group** | **Preen lie** | **Lie/Rest** | **Lie eat** | **Lie sleep** | **Leg stretch** | **Wing stretch** | **Leg/wing stretch** | **Wing flap** | **Total** |
| --- | --- | --- | --- | --- | --- | --- | --- | --- | --- | --- |
| **1** | **Control** | 3.3 | 7.9 | 2.0 | --- | 1.3 | 2.3 | 2.0 | 1.7 | 20.5±0.8 |
| **2** | **Probiotic** | 3.4 | 6.9 | --- | 1.2 | 1.5 | 1.9 | 2.3 | 2.8 | 20±0.7 |
| **3** | **IgY** | 3.8 | 6.9 | 1.6 | 3.1 | --- | 2.6 | 1.6 | 2.1 | 21.7±0.6 |
| **4** | **Probiotic + IgY** | 2.8 | 7.3 | 3.4 | 2.3 | --- | 1.9 | 2.3 | 2.1 | 22.1±0.7 |

The mean frequency of behavioural patterns such as rest/lying (e.g., preen lie, lie, lie eat, lie sleep, leg stretch, wing stretch, leg and wing stretch, wing flap) were recorded and analyzed using one-way-ANOVA. lying frequency was presented as mean ± SEM. IgY: immunoglobulin Y; (---): not detected.

**TABLE S5** The statistics of standing activity time (sec/ 30 min) at 4^th^ week of broilers supplemented with variant feed additives.

| **No.** | **Group** | **Preen stand** | **Stand** | **Stand eat** | **Peck body** | **Peck head** | **Peck litter** | **Drink** | **Walk** | **Jump** | **Run** | **Total** |  |
| --- | --- | --- | --- | --- | --- | --- | --- | --- | --- | --- | --- | --- | --- |
| **1** | **Control** | --- | 24.7 | 255.3 | 2.2 | --- | 3.1 | 189.3 | 41.1 | --- | --- | 515.7±34.3 | |
| **2** | **Probiotic** | --- | 3.5 | 86.0 | 1.0 | --- | 1.7 | 94.2 | 14.9 | --- | --- | 201.3±13.9 | |
| **3** | **IgY** | --- | 48.5 | 371.8 | --- | --- | --- | 81.8 | 41.9 | --- | --- | 544±50.01 | |
| **4** | **Probiotic + IgY** | --- | 61.5 | 606.8 | 125 | --- | 200 | 166.1 | 183.8 | --- | --- | 1343.2±61.4* | |
| The meantime of behavioural patterns such as standing activity (e.g., preen stand, stand, stand eat, peck body, peck head, peck litter, drink, walk, jump and run) were recorded and analyzed using one-way-ANOVA. Standing activity was presented as mean ± SEM. IgY: immunoglobulin Y. (*), *P*< 0.05; (---): not detected. | | | | | | | | | | | | | |

**TABLE S6** The statistics of lying time (sec/ 30 min) at 4^th^ week of broilers supplemented with variant feed additives.

| **No.** | **Group** | **Preen lie** | **Lie/Rest** | **Lie eat** | **Lie sleep** | **Leg stretch** | **Wing stretch** | **Leg/wing stretch** | **Wing flap** | **Total** |
| --- | --- | --- | --- | --- | --- | --- | --- | --- | --- | --- |
| **1** | **Control** | 147.3 | 684.1 | 378.2 | 198.9 | 4.2 | 1.4 | --- | --- | 1414.1±92.06 |
| **2** | **Probiotic** | 129.2 | 246.7 | 299.4 | 101.2 | 2.9 | 0.2 | --- | --- | 779.6±43.6 |
| **3** | **IgY** | 49.8 | 1147.6 | 44.0 | --- | --- | --- | 4.1 | --- | 1245.5±197.2 |
| **4** | **Probiotic + IgY** | 59 | 361.6 | 34.0 | --- | --- | --- | 2.1 | --- | 456.7±58.8* |
| The meantime of behavioural patterns such as rest/lying (e.g., preen lie, lie, lie eat, lie sleep, leg stretch, wing stretch, leg and wing stretch, wing flap) were recorded and analyzed using one-way-ANOVA. Lying time was presented as mean ± SEM. IgY: immunoglobulin Y; (*), P< 0.05; (---): not detected. | | | | | | | | | | |

**TABLE S7** The statistics of frequency of activity (bout/ 30 min) at 4^th^ week of broilers supplemented with variant feed additives.

| **No.** | **Group** | **Preen stand** | **Stand** | **Stand eat** | **Peck body** | **Peck head** | **Peck litter** | **Drink** | **Walk** | **Jump** | **Run** | **Total** |
| --- | --- | --- | --- | --- | --- | --- | --- | --- | --- | --- | --- | --- |
| **1** | **Control** | --- | 1.9 | 2.0 | 2.1 | --- | 1.6 | 3.8 | 5.0 | --- | --- | 16.4±0.4 |
| **2** | **Probiotic** | 1.5 | 5.7 | --- | --- | --- | --- | 3.9 | 9.8 | --- | --- | 20.9±1.1 |
| **3** | **IgY** | 4 | 6 | 3 | 4 | --- | --- | 5.45 | 5 | --- | --- | 27.45±0.3 |
| **4** | **Probiotic + IgY** | 3 | 5 | 4 | 3 | --- | 1 | 4.48 | 5 | --- | --- | 25.48±0.4 |
| The mean frequency of behavioural patterns such as standing activity (e.g., preen stand, stand, stand eat, peck body, peck head, peck litter, drink, walk, jump and run) were recorded and analyzed using one-way-ANOVA. Standing activity frequency was presented as mean ± SEM. IgY: immunoglobulin Y; (---): not detected. | | | | | | | | | | | | |

**TABLE S8** The statistics of frequency of lying (bout/ 30 min) at 4^th^ week of broilers supplemented with variant feed additives.

| **No.** | **Group** | **Preen lie** | **Lie/Rest** | **Lie eat** | **Lie sleep** | **Leg stretch** | **Wing stretch** | **Leg/wing stretch** | **Wing flap** | **Total** |
| --- | --- | --- | --- | --- | --- | --- | --- | --- | --- | --- |
| **1** | **Control** | 2.8 | 5.2 | 1.7 | --- | --- | 1.2 | --- | --- | 10.9±0.6 |
| **2** | **Probiotic** | 3.9 | 7.6 | --- | --- | --- | 1.3 | 2.5 | --- | 15.3±0.9 |
| **3** | **IgY** | 3 | 7 | 1.07 | --- | 1 | 1 | 2 | --- | 15.07±0.8 |
| **4** | **Probiotic + IgY** | 2 | 6 | 1 | --- | 2 | 1.6 | --- | --- | 12.6±0.7 |
| The mean frequency of behavioural patterns such as rest/lying (e.g., preen lie, lie, lie eat, lie sleep, leg stretch, wing stretch, leg and wing stretch, wing flap) were recorded and analyzed using one-way-ANOVA. Lying frequency was presented as mean ± SEM. IgY: immunoglobulin Y; (---): not detected. | | | | | | | | | | |

**TABLE S9** The statistics of standing activity time (sec/ 30 min) at 5^th^ week of broilers supplemented with variant feed additives.

| **No.** | **Group** | **Preen stand** | **Stand** | **Stand eat** | **Peck body** | **Peck head** | **Peck litter** | **Drink** | **Walk** | **Jump** | **Run** | **Total** |
| --- | --- | --- | --- | --- | --- | --- | --- | --- | --- | --- | --- | --- |
| **1** | **Control** | --- | 39.3 | 284.3 | 48.2 | --- | 43.2 | 145.1 | 35.2 | --- | --- | 595.3±31.5 |
| **2** | **Probiotic** | 39.9 | 77.0 | 404.4 | 23.2 | 13.0 | 15.5 | 138.3 | 32.8 | --- | 7.3 | 751.4±40.2 |
| **3** | **IgY** | 15.8 | 72.9 | 261.8 | --- | --- | 39.2 | 200.4 | 66.6 | --- | 18.4 | 675.1±30.4 |
| **4** | **Probiotic + IgY** | 19.2 | 67.4 | 291.1 | 7.3 | --- | 31.0 | 146.6 | 62.1 | --- | --- | 624.7±31.6** |
| The meantime of behavioural patterns such as standing activity (e.g., preen stand, stand, stand eat, peck body, peck head, peck litter, drink, walk, jump and run) were recorded and analyzed using one-way-ANOVA. Standing activity was presented as mean ± SEM. IgY: immunoglobulin Y. (**), P< 0.01. (---): not detected. | | | | | | | | | | | | |

**TABLE S10** The statistics of lying time (sec/ 30 min) at 5^th^ week of broilers supplemented with variant feed additives.

| **No.** | **Group** | **Preen lie** | **Lie/Rest** | **Lie eat** | **Lie sleep** | **Leg stretch** | **Wing stretch** | **Leg/wing stretch** | **Wing flap** | **Total** |
| --- | --- | --- | --- | --- | --- | --- | --- | --- | --- | --- |
| **1** | **Control** | 43.0 | 923.0 | 48.1 | 202.3 | --- | 4.5 | 6.8 | --- | 1227.7±127.07 |
| **2** | **Probiotic** | 30.2 | 631.4 | 159.5 | --- | --- | 3.6 | 3.7 | 2.4 | 830.8±88.03 |
| **3** | **IgY** | 17.0 | 802.7 | 123.4 | 180.7 | --- | 6.5 | 6.7 | --- | 1137±109.2 |
| **4** | **Probiotic + IgY** | 36.7 | 585.2 | 104.8 | --- | 6.2 | 3.8 | --- | 3.8 | 740.5±81.1 |
| The meantime of behavioural patterns such as rest/lying (e.g., preen lie, lie, lie eat, lie sleep, leg stretch, wing stretch, leg and wing stretch, wing flap) were recorded and analyzed using one-way-ANOVA. Lying time was presented as mean ± SEM. IgY: immunoglobulin Y. (---): not detected. | | | | | | | | | | |

**TABLE S11** The statistics of frequency of activity (bout/ 30min) at 5^th^ week of broilers supplemented with variant feed additives.

| **No.** | **Group** | **Preen stand** | **Stand** | **Stand eat** | **Peck body** | **Peck head** | **Peck litter** | **Drink** | **Walk** | **Jump** | **Run** | **Total** |
| --- | --- | --- | --- | --- | --- | --- | --- | --- | --- | --- | --- | --- |
| **1** | **Control** | --- | 3.3 | 3.1 | 3.1 | --- | 1.9 | 4.8 | 5.0 | --- | --- | 21.2±0.37 |
| **2** | **Probiotic** | 2.6 | 4.1 | 4.1 | 2.8 | --- | 2.1 | 4.5 | 6.6 | --- | 1.5 | 28.3±0.51 |
| **3** | **IgY** | 3.0 | 5.9 | 4.1 | --- | --- | 3.3 | 3.1 | 9.8 | --- | 2.4 | 31.6±0.81** |
| **4** | **Probiotic + IgY** | 3.3 | 3.3 | 4.0 | 2.5 | --- | 4.3 | 3.1 | 6.3 | --- | --- | 26.8±0.39 |
| The mean frequency of behavioural patterns such as standing activity (e.g., preen stand, stand, stand eat, peck body, peck head, peck litter, drink, walk, jump and run) were presented as mean ± SEM and analyzed using one-way-ANOVA. IgY: immunoglobulin Y. (**), *P*< 0.01; (---): not detected. | | | | | | | | | | | | |

**TABLE S12**The statistics of frequency of lying (bout/ 30min) at 5^th^ week of broilers supplemented with variant feed additives.

| **No.** | **Group** | **Preen lie** | **Lie/Rest** | **Lie eat** | **Lie sleep** | **Leg stretch** | **Wing stretch** | **Leg/wing stretch** | **Wing flap** | **Total** |
| --- | --- | --- | --- | --- | --- | --- | --- | --- | --- | --- |
| **1** | **Control** | 2.8 | 7.2 | 1.6 | 1.8 | --- | 1.8 | 2.0 | --- | 17.2±0.76 |
| **2** | **Probiotic** | 3.9 | 6.6 | 1.9 | --- | --- | 1.7 | 1.0 | --- | 15.1±0.80 |
| **3** | **IgY** | 2.5 | 6.6 | 3.3 | 1.8 | --- | 1.8 | 2.1 | --- | 18.1±0.65 |
| **4** | **Probiotic + IgY** | 4.1 | 6.9 | 1.9 | --- | 1.1 | 1.6 | --- | --- | 15.6±0.85 |
| The mean frequency of behavioural patterns such as rest/lying (e.g., preen lie, lie, lie eat, lie sleep, leg stretch, wing stretch, leg and wing stretch, wing flap) were recorded and analyzed using one-way-ANOVA. Lying frequency was presented as mean ± SEM. IgY: immunoglobulin Y; (---): not detected. | | | | | | | | | | |

**TABLE S13** The statistics of standing activity time (sec/ 30min) at 6^th^ week of broilers supplemented with variant feed additives.

| **No.** | **Group** | **Preen stand** | **Stand** | **Stand eat** | **Peck body** | **Peck head** | **Peck litter** | **Drink** | **Walk** | **Jump** | **Run** | **Total** |
| --- | --- | --- | --- | --- | --- | --- | --- | --- | --- | --- | --- | --- |
| **1** | **Control** | --- | 11.7 | 311.0 | 7.0 | 2.7 | 91.0 | 137.3 | 21.9 | --- | 3.1 | 585.7±108.004 |
| **2** | **Probiotic** | 14.7 | 96.2 | --- | 24.9 | --- | 78.4 | 136.3 | 26.3 | --- | --- | 376.8±48.6 |
| **3** | **IgY** | 9.6 | 63.0 | 276.4 | 8.4 | --- | 105.6 | 98.9 | 26.6 | --- | 5.6 | 594.1±91.08 |
| **4** | **Probiotic + IgY** | --- | 55.8 | 229.4 | 9.9 | --- | 184.2 | 216.0 | 18.5 | --- | --- | 713.8±101.83** |
| The meantime of behavioural patterns such as standing activity (e.g., preen stand, stand, stand eat, peck body, peck head, peck litter, drink, walk, jump and run) were recorded and analyzed using one-way-ANOVA. Standing activity time was presented as mean ± SEM. IgY: immunoglobulin Y. (**) P < 0.01; (---): not detected. | | | | | | | | | | | | |

**TABLE S14** The statistics of lying time (sec/ 30min) at 6^th^ week of broilers supplemented with variant feed additives.

| **No.** | **Group** | **Preen lie** | **Lie/Rest** | **Lie eat** | **Lie sleep** | **Leg stretch** | **Wing stretch** | **Leg/wing stretch** | **Wing flap** | **Total** |
| --- | --- | --- | --- | --- | --- | --- | --- | --- | --- | --- |
| **1** | **Control** | 32.6 | 1047.1 | 479.7 | --- | 97.0 | 4.1 | 12.8 | 5.0 | 1678.3±139.55 |
| **2** | **Probiotic** | 46.9 | 828.0 | 286.3 | --- | 47.7 | 6.0 | 10.1 | 1.9 | 1226.9±107.73 |
| **3** | **IgY** | 49.8 | 844.3 | 241.1 | --- | 58.4 | 4.3 | 10.1 | 3.6 | 1211.6±108.71 |
| **4** | **Probiotic + IgY** | 31.4 | 696.6 | 189.8 | --- | --- | --- | --- | 3.9 | 921.7±113.63** |
| The meantime of behavioural patterns such as rest/lying (e.g., preen lie, lie, lie eat, lie sleep, leg stretch, wing stretch, leg and wing stretch, wing flap) were recorded and analyzed using one-way-ANOVA. Lying time was presented as mean ± SEM. IgY: immunoglobulin Y. (**) *P*< 0.01; (---): not detected. | | | | | | | | | | |

**TABLE S15** The statistics of frequency of activity (bout/ 30min) at 6^th^ week of broilers supplemented with variant feed additives.

| **No.** | **Group** | **Preen stand** | **Stand** | **Stand eat** | **Peck body** | **Peck head** | **Peck litter** | **Drink** | **Walk** | **Jump** | **Run** | **Total** |
| --- | --- | --- | --- | --- | --- | --- | --- | --- | --- | --- | --- | --- |
| **1** | **Control** | --- | 1.6 | 2.0 | 1.5 | 1.0 | 3.8 | 6.5 | 6.0 | --- | 1.3 | 23.7±0.69 |
| **2** | **Probiotic** | 4.2 | 4.2 | --- | 2.1 | --- | 4.8 | 6.9 | 7.6 | --- | --- | 29.8±0.63 |
| **3** | **IgY** | 2.4 | 2.5 | 3.2 | 1.5 | --- | 5.4 | 9.5 | 8.5 | --- | 2.0 | 35±0.97 |
| **4** | **Probiotic + IgY** | --- | 4.1 | 1.9 | 1.8 | --- | 4.6 | 8.0 | 7.1 | --- | --- | 27.5±0.81 |
| The mean frequency of behavioural patterns such as standing activity (e.g., preen stand, stand, stand eat, peck body, peck head, peck litter, drink, walk, jump and run) were recorded and analyzed using one-way-ANOVA. Standing activity frequency was presented as mean ± SEM. IgY: immunoglobulin Y; (---): not detected. | | | | | | | | | | | | |

**TABLE S16** The statistics of frequency of lying (bout/ 30min) at 6^th^ week of broilers supplemented with variant feed additives.

| **No.** | **Group** | **Preen lie** | **Lie/Rest** | **Lie eat** | **Lie sleep** | **Leg stretch** | **Wing stretch** | **Leg/wing stretch** | **Wing flap** | **Total** |
| --- | --- | --- | --- | --- | --- | --- | --- | --- | --- | --- |
| **1** | **Control** | 4.2 | 7.1 | 2.6 | --- | 1.3 | 1.9 | 2.0 | 1.8 | 20.9±0.72 |
| **2** | **Probiotic** | 4.5 | 7.9 | 2.7 | --- | 1.7 | 1.9 | 1.4 | 1.2 | 21.3±0.85 |
| **3** | **IgY** | 5.1 | 8.5 | 3.2 | --- | 2.7 | 1.9 | 1.4 | 1.9 | 24.7±0.88 |
| **4** | **Probiotic + IgY** | 4.8 | 6.1 | 2.9 | --- | --- | --- | --- | 1.8 | 15.6±0.67 |
| The mean frequency of behavioural patterns such as rest/lying (e.g., preen lie, lie, lie eat, lie sleep, leg stretch, wing stretch, leg and wing stretch, wing flap) were recorded and analyzed using one-way-ANOVA. Lying frequency was presented as mean ± SEM. IgY: immunoglobulin Y; (---): not detected. | | | | | | | | | | |

**TABLE S17** Calculation of mortality% of chicken broilers (n=15/each group) during the experiment.

| **No.** | **Group** | **Day** | **Dead** | **Mortality%** |
| --- | --- | --- | --- | --- |
| **1** | **Control-saline** | 7 | 1 | 13 |
|  |  | 34 | 1 |  |
| **2** | **Probiotic** | --- | --- | 0 |
| **3** | **IgY** | --- | --- | 0 |
| **4** | **Probiotic + IgY** | --- | --- | 0 |
| IgY: immunoglobulin. (---): not detected. | | | | |
